# Acute non-ambulatory tetraparesis with absence of the dens in two large breed dogs: case reports with a radiographic study of relatives

**DOI:** 10.1186/1751-0147-55-31

**Published:** 2013-04-17

**Authors:** Øyvind Stigen, Mona Aleksandersen, Randi Sørby, Hannah J Jørgensen

**Affiliations:** 1 Department of Companion Animal Clinical Sciences, Norwegian School of Veterinary Science, PO Box 8146, Oslo, NO-0033, Norway; 2 Department of Basic Sciences, Norwegian School of Veterinary Science, PO Box 8146, Oslo, NO-0033, Norway; 3 Norwegian Veterinary Institute, PO Box 750, Oslo, NO-0106, Norway

**Keywords:** Dens, Atlantoaxial subluxation, Tetraparesis, Dog, Large-breed

## Abstract

**Background:**

Non-ambulatory tetraparesis with an absence of the dens of C2 (axis) has not previously been reported in large breed dogs. An absence or hypoplasia of the dens has been reported in both small, medium and large breed dogs, but not in closely related animals.

**Methods:**

Two young large-breed dogs (a German shepherd and a Standard poodle) both with an acute onset of non-ambulatory tetraparesis were subjected to physical, neurological and radiographic examinations. Both dogs were euthanased and submitted for postmortem examination within one week of onset of clinical signs. To investigate possible heritability of dens abnormalities, oblique radiographs of the cranial cervical vertebrae were taken of nine and eighteen dogs related to the German shepherd and the Standard poodle, respectively.

**Results:**

Absence of the dens, atlantoaxial instability and extensive spinal cord injury was found in both case dogs. Radiographs revealed a normal dens in both parents and in the seven littermates of the German shepherd. An absence or hypoplasia of the dens was diagnosed in six relatives of the Standard poodle.

**Conclusions:**

Atlantoaxial subluxation with cervical spinal cord injury should be considered as a differential diagnosis in non-ambulatory tetraparetic young large breed dogs. Absence of the dens and no history of external trauma increase the likelihood for this diagnosis. This study provides evidence to suggest that absence or hypoplasia of the dens is inherited in an autosomal way in Standard poodle dogs.

## Background

Atlantoaxial subluxation, although uncommon, may be a cause of neck pain and neurological deficits in the dog. The condition can be traumatic, congenital or developmental in origin. The traumatic form affects dogs of any size, age or breed whereas the congenital and developmental forms usually occur in toy breed dogs younger than one year of age [1-3]. Most clinical cases are result from a combination of congenital or developmental and traumatic causes. Congenital and developmental anomalies predispose the animal to subluxation by relatively minor trauma [4].

Congenital and developmental lesions seen with atlantoaxial subluxation are aplasia (absence) or hypoplasia of the dens [1,3,5-7], dorsal angulation of the dens [1,8-11], non-union of the dens with the vertebral body of the axis [7,12], absence of the transverse ligament of the atlas [13] and incomplete ossification of the atlas [14,15]. Regardless of the type of predisposing lesion, only dorsal subluxation has been reported. On lateral radiographs this is demonstrated by an increased space between the dorsal arch of the atlas (C1) and the spinous process of the axis (C2) [4,16]. A dynamic nature of the subluxation has also been demonstrated, where mild flexion of the neck was necessary to provoke the malalignment of the vertebra [16,17].

The dens of the axis has an important role in maintaining stability of the atlantoaxial joint by its attachments to the occipital bone with the apical and bilateral alar ligaments, and its binding to the floor of C1 with the transverse ligament of the atlas [18]. An absence or hypoplasia of the dens represents a significant reduction of the C1-C2 intervertebral stability. Not surprisingly one of these two conditions is found in 46–58% of dogs with clinically significant atlantoaxial subluxation [2,3].

Atlantoaxial subluxation with absence or hypoplasia of the dens has been reported in eight large- or medium-sized dog breeds including the Doberman [19-21], Basset Hound [22], Weimaraner [10], Saint Bernard [23] and Rottweiler [24,25]. With the exception of the Saint Bernard dog, all the cases presented with neck pain and generalized ataxia. None of the seven dogs showed non-ambulatory tetraparesis or were euthanased because of severe cervical cord injury. Watson *et al.*[23] reported a thirteen-week-old Saint Bernhard with a five week history of tetraplegia. This dog was diagnosed with a congenital occipito-atlanto-axial malformation (including hypoplasia and deviation of the dens) and was euthanased.

The present paper reports two cases of acute non-ambulatory tetraparesis caused by spinal cord injury at C1-C2 in large breed dogs (one German shepherd and one Standard poodle). In both cases, the dens was absent and atlantoaxial instability was found at postmortem examinations. A radiographic examination of parents, littermates and offspring of the two dogs revealed an absent or hypoplastic dens in six of the examined Standard poodle dogs. None of these showed signs of neck pain or gait disturbances on the day of radiographic examination. This is the first paper to describe non-ambulatory tetraparesis with absence of the dens in large breed dogs and to document aplasia/hypoplasia of the dens in closely related dogs.

## Case presentations

### Dog 1

A nine-month-old, 26 kg, female German shepherd dog, was referred to the small animal clinic at the Norwegian School of Veterinary Science (NVH) for further evaluation of non-ambulatory tetraparesis. The condition had occurred eighteen hours prior to admission. According to the owner, the dog had been playing with a ball and was shaking its head when it suddenly yelped and collapsed. The owner also described «a blue-coloured tongue», indicating hypoxia. At immediate clinical examination, the referring veterinarian observed a confused dog that made unsuccessful attempts to stand up. It showed no signs of pain, but muscle tone was increased in all four limbs. The tongue and mucous membranes were normal. The dog was treated empirically with 20 mg xylazine and 35 mg prednisolone intramuscularly.

At admission, the dog was in normal body condition, bright and alert, but lay in lateral recumbency with a total absence of voluntary motor function in all four limbs (tetraplegia). It was able to partly raise and move its head. The rectal body temperature was 39.9°C, and the heart rate was 80/min. On neurological examination, cranial nerve function was normal. There was increased muscle tone in the right thoracic and pelvic limbs. Deep pain perception was present and spinal cord reflexes were considered to be exaggerated in all four limbs. A crossed extensor reflex was observed in both pelvic limbs. On moderate manipulation of the neck, the dog showed no signs of pain. Abdominal palpation revealed an enlarged urinary bladder.

Further diagnostic evaluation included biochemical analysis and a complete blood count. All results were within the reference ranges for the laboratory. Urine was gently expressed, and urinalysis (mid-stream urine) revealed a specific gravity of 1.040 (reference range 1.030 to 1.065) and 3+ (50 Ery/μl) blood on test strip analysis (Kruuse, Langeskov, Denmark). Microscopy of urine sediment revealed high numbers of erythrocytes and nucleated and non-nucleated epithelial cells. Urine culture was not performed.

**Figure 1:**
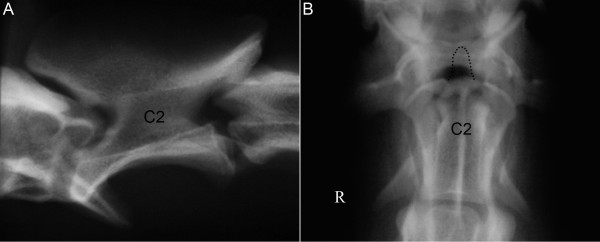
**Lateral (A) and ventrodorsal (B) radiographs of the cranial cervical vertebrae of dog 1.** An absence of the dens of C2, but no signs of atlantoaxial subluxation. The broken line inserted on the ventrodorsal view (B) indicates the border for a normal dens.

The neurological examination localized the lesion to the C1-C5 spinal cord segment. Plain lateral and ventrodorsal radiographs of the cervical spine showed an absence of the dens of C2, but no signs of atlantoaxial subluxation (Figure 1). A lateral view with mild flexion of the neck was not performed. Based on the clinical history, and the neurological and radiographical examinations, a severe spinal cord injury to the C1 to C5 spinal cord segment as result of an acute and temporary atlantoaxial subluxation was suspected. The absence of the dens was suspected to be the primary cause of an atlantoaxial instability and subsequent subluxation of the joint.

The dog was treated with iv lactated Ringer´s solution, 20 mg/kg ampicillin (Pentrexyl; Bristol-Myers Squibb AB, Bromma, Sweden) iv twice daily and 4 mg/kg carprofen (Rimadyl; Pfizer, New York, USA) iv once daily. The body temperature was measured twice daily and varied between 39.2 and 40.6°C. After three days, no improvement in neurological signs was observed. The prognosis for full neurological recovery was considered to be poor and with the owner´s consent the dog was euthanased and submitted for postmortem examination.

**Figure 2:**
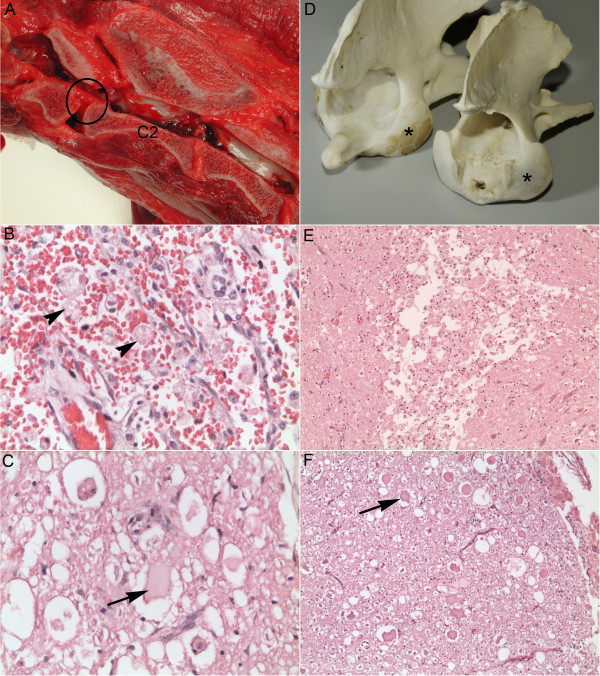
**Macro- and microscopic findings, left panel from dog 1, and right panel from dog 2.** (**A**) Sagittal section of the neck of dog 1 showing an absence of the dens (encircled area) of C2. (**B**) Grey matter lesions of dog 1 showing haemorrhage and numerous gitter cells (arrow heads). (**C**) White matter lesions in the ventral funiculi of dog 1 showing numerous spheroids (arrow) and dilated myelin sheaths with macrophages. (**D**) Axis from dog 2 (right) compared to axis from a normal, five-year-old boxer dog (left). In dog 2, the dens is absent and an irregular depressed area is present in the normal position of the dens. The cranial articular surfaces (asterisks) in dog 2 are smaller than in the normal dog. (**E**) Grey matter lesions of dog 2 showing an area of necrosis with gitter cells. (**F**) White matter lesions of dog 2 showing numerous spheroids (arrow) and dilated myelin sheaths. All histological sections shown (B,C,E,F) are from the atlantoaxial area of the spinal cord.

Gross examination showed increased mobility of the cranial part of the cervical columna. A sagittal section through the cervical columna revealed that the dens at C2 was absent (Figure 2A). The spinal cord in this area was discoloured with a greyish red surface and a darker, brown cut surface.

Microscopical examination showed subdural haemorrhage dorsal to the spinal cord at the atlantoaxial level. The spinal cord in this area showed extensive necrotic and degenerative lesions. The most severe lesions were found dorsolateral to the central canal and the zone between grey and white matter was difficult to identify. Multifocal to diffuse haemorrhages were present in grey and white matter of the cervical spinal cord (Figure 2B). The white matter of the dorsal funiculi were severely affected and showed diffuse degeneration and focal necrosis. The white matter of the lateral and ventral funiculi showed degenerative lesions with numerous spheroids and dilated myelin sheaths with presence of macrophages (Figure 2C). The grey matter had extensive necrosis with loss of neuropil and neurons and presence of numerous gitter cells (Figure 2B). The cervical spinal cord caudal to C2 had moderate degenerative lesions in white matter of lateral and ventral funiculi. Some dilated myelin sheaths were present in the nerve roots.

### Dog 2

A twelve-month-old female Standard poodle dog, weighing 23 kg, was referred to the NVH for evaluation of non-ambulatory tetraparesis with a duration of seven days. The dog had been playing outdoors when it suddenly collapsed and lost consciousness. According to the owner, the dog´s condition worsened and it suffered serious respiratory failure during transit to the referring veterinarian. At the referring small animal clinic the dog was stabilised with oxygen and iv lactated Ringer´s solution. The following day a neurological examination was performed. The examination revealed a somewhat depressed dog in lateral recumbency. There were obvious voluntary movements in all four limbs, but the dog could not change position or stand without considerable assistance. The dog could raise its head and freely move it. Dorsal, lateral and ventral flexion of the neck did not cause any signs of pain. Signs of cranial nerve dysfunction were not registered. Based on the clinical history and the clinical signs a cervical fibrocartilaginous embolic myelopathy was suspected and treatment with medical and supportive care was prescribed for the coming days.

**Figure 3:**
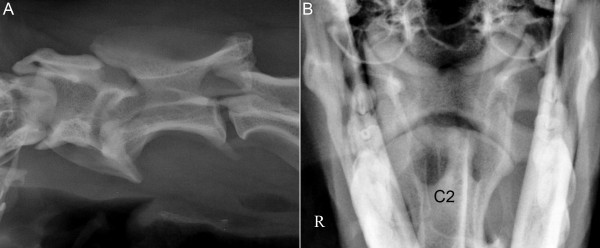
**Oblique lateral (A) and ventrodorsal open-mouth (B) radiographs of the cranial cervical vertebrae of dog 2.** An absence of the dens of C2, but no signs of atlantoaxial subluxation.

Upon arrival at the NVH, the dog was still in lateral recumbency and had voluntary movements in all four limbs. Body temperature was 39.1°C. The neurological deficits noted at the preliminary examination were still present. Furthermore, an increased muscle tone and hyperreflexia were observed in all four limbs and a crossed extensor reflex was observed in both pelvic limbs. Signs of neck pain were absent and deep pain perception was present in all four limbs. The neurological examination indicated a C1-C5 spinal cord lesion. Oblique lateral and ventrodorsal open-mouth radiographs of the cervical spine revealed an absence of the dens of C2 (Figure 3), but malalignment of the bodies of the atlas and axis was not visible. A flexed lateral view was not performed. The cervical cord lesion was presumed to be the result of an acute and temporary atlantoaxial subluxation related to the absence of dens.

Owing to the severity of the neurological deficits and the clinical history, including no signs of improvement over seven days, the prognosis was considered to be guarded. Surgical stabilization of the atlantoaxial joint was offered, but the owner declined and elected euthanasia.

At necropsy the dens was confirmed to be absent. A round irregularly depressed area of 0.5 × 0.5 cm was noted in the normal position of the dens (Figure 2D). The left and right cranial articular surface of the axis were smaller than normal. The spinal cord was moderately flattened dorsoventrally in the C1 to C2 segment, however haemorrhages or yellow discoloured areas (malacia) were not noted macroscopically.

Microscopically, in the C1 to C2 segment of the spinal cord, there were multifocal degenerative and necrotizing lesions. In grey matter there were foci of necrosis with many gitter cells (Figure 2E), and in some sections these foci were bilaterally symmetric in dorsolateral position to the central canal. White matter was extensively affected with numerous dilated myelin sheaths and spheroids (Figure 2F). The lesions were severe in the lateral and ventral funiculi, and mild in the dorsal funiculi. Moderate numbers of macrophages were present in the dilated myelin sheaths. The brain and other segments of the spinal cord in other areas were unremarkable.

### Radiographic study of related dogs

Parents and littermates (full siblings) of dog 1 and 2 were identified with support from the breeders. The owners of these dogs were invited to present their dogs for a physical and radiographic examination either at the NVH or at their local small animal clinic. In total, two parents and seven littermates of dog 1 and one parent and seven littermates of dog 2 were available for examination. The dogs that were unavailable for examination were either dead (2 dogs) or the owners did not respond on the request (2 dogs). The causes of death of the two dogs were reported to be umbilical bleeding and intestinal foreign body, respectively.

Oblique radiographs of the cranial cervical vertebrae were taken and the presence, size and shape of the dens was evaluated according to previously described methods of examination [26-28]. A ventrodorsal view with the neck extended was included in dogs that showed dens abnormalities on the oblique view.

**Figure 4:**
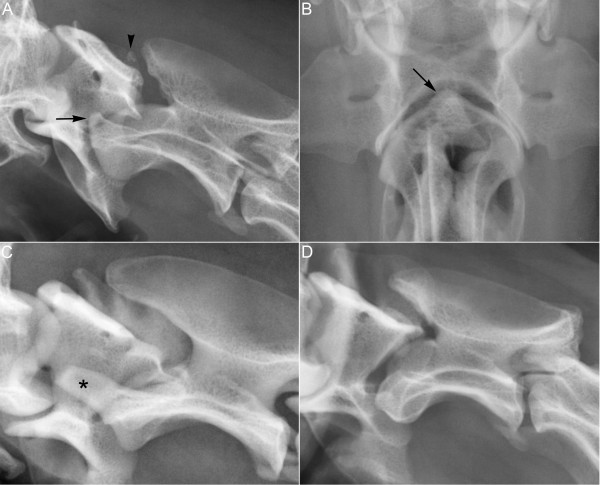
**Radiographs of the cranial cervical vertebrae of three Standard poodle dogs related to dog 2.** The oblique lateral **(A)** and the ventrodorsal **(B)** views of the mother of dog 2, demonstrates a severe degree of dens hypoplasia (arrows). The oblique lateral view (A) also reveals a calcified body (arrowhead) between the dorsal arch of C1 and the spinous process of C2. The oblique lateral view of a brother of dog 2 **(C)** shows a normal dens (asterisk) whereas the oblique lateral view of a half sister of dog 2 **(D)** shows an absence of the dens.

A normal dens (Figure 4C) was identified in both parents and in all seven littermates of dog 1. A severe degree of dens hypoplasia was found in the parent (mother) of dog 2 (Figure 4A and B), and the dens was found to be absent in one littermate (brother) of dog 2. The oblique radiograph taken of the eight-year-old mother, showed that the alignment of C1 and C2 was questionable and a calcified body was seen between the dorsal arch of the C1 and the spinous process of the C2 (Figure 4A). The calcified body was interpreted as focal dystrophic calcification at the dorsal atlantoaxial ligament. No signs of neck pain or gait disturbances were detected on the physical examination of any of the parents or littermates. None of the owners reported previous signs of neck pain or gait disturbances in their dogs.

Subsequently, the owner of a three-year-old female Standard poodle dog, that was the offspring of a previous litter of the mother of dog 2, contacted the corresponding author. She reported that two years previously her dog (then nine-month-old) had suddenly yelped and fallen over when it was running around at a field. The dog lay on the ground for one hour before it managed to stand up and walk unsteadily. The local veterinarian diagnosed an ambulatory tetraparesis and prescribed rest and 20 mg prednisolone orally once daily for ten days. The dog gradually improved and two weeks later no gait disturbances were seen. Neck pain had never been registered. For this dog, a corresponding physical and radiographic examination was agreed and was performed at the NVH. No abnormalities were detected at the physical examination, but on the oblique (Figure 4D) and ventrodorsal radiographs the dens was found to be absent.

The owner of this three-year-old female poodle reported that her dog had given birth to nine puppies six months previously, and that the present owner of one of these puppies had observed episodic seizures with an absence of motor function in all four limbs in his dog. The owners of all the nine now six-month-old dogs were invited to present their animals for a physical and radiographic examination. All nine dogs were presented and at the physical examination no signs of neck pain or gait disturbances were detected in any of them. The radiographic examination revealed a normal dens in six dogs, an absence of the dens in two (one male and one female), and hypoplasia of the dens in one (male). The dog with the history of episodic seizures, was the male dog with an absent dens.

## Discussion

The present paper documents that atlantoaxial subluxation due to absence of the dens can cause an acute non-ambulatory tetraparesis in two different breeds of large dogs. The cases described herein showed no sign of neck pain, hence, an absence of such pain is compatible with the diagnosis. Previous reports describe neck pain, ataxia and ambulatory tetraparesis as symptoms with the congenital or developmental form of atlantoaxial subluxation in large-breed dogs [10,20,22,24,25]. Thus, clinical signs associated with this condition in large dogs can vary considerably.

In this report, we describe one German shepherd (Dog 1) and three poodles that presented with an absent dens and acute non-ambulatory tetraparesis during the first year of life. However, signs of neck pain or gait disturbances were not detected in four older poodle dogs with an absence or hypoplasia of the dens. This indicates that large breed dogs without a dens run the greatest risk of severe atlantoaxial subluxation at an early age. In adult age, hypertrophy of atlantoaxial muscles, tendons, ligaments and the joint capsule may stabilize the joint to the extent that clinical signs of subluxation do not appear. A similar theory is presented by Patton *et al.*[29], who reported a rottweiler dog with a nine year history of mild neurological deficits, an absent dens, but no associated displacement between the atlas and axis. It is speculated that in large breed dogs, the large neck muscle mass together with a thickening of the atlantoaxial joint capsule and the dorsal atlantoaxial ligament, can prevent atlantoaxial instability [29].

In dogs 1 and 2, atlantoaxial instability and pathological changes in the cervical cords were found at the postmortem examinations. Although neck pain is a frequent finding in dogs with atlantoaxial subluxation, it was not registered at the clinical examinations. Immediate repositioning of the atlantoaxial subluxation, cage rest and treatment with analgesics probably neutralized pain arising from articular joint capsules in both of these dogs. Another possible explanation for the apparent absence of neck pain is that the pathological changes were limited to spinal cord tissue, and insignificantly affected pain sensitive structures such as meninges, nerve roots, intervertebral discs or periosteum. In toy breed dogs with atlantoaxial subluxation, neck pain is reported to occur in 30–60% [1,3].

The lateral and ventrodorsal radiographs of dogs 1 and 2 showed no signs of atlantoaxial subluxation, but increased atlantoaxial mobility was found in both dogs at the postmortem examinations. Most likely, subluxation would have been demonstrated radiographically in both dogs if flexed lateral view radiographs had been taken. However, this 'stressed' position may cause further spinal cord injury and, therefore, was not included as part of the diagnostic procedures. However, flexed lateral view radiographs could have been taken after euthanasia, as part of the post-mortem examinations.

The calcified body that was seen on the oblique view in the mother of dog 2 was interpreted as dystrophic calcification, and is most likely the result of a stress-induced injury to the dorsal atlantoaxial ligament. In dogs with subluxation of the atlantoaxial joint a total rupture of the dorsal atlantoaxial ligament, caused by hyperflexion of the head, is commonly registered [4,30]. In the mother of dog 2 we presume that the dorsal atlantoaxial ligament has been partially ruptured by constant atlantoaxial instability. The severe degree of dens hypoplasia and the questionable alignment of C1 and C2 are also in accordance with presence of instability. This case shows that a discrepancy between radiological findings and clinical signs may exist in dogs with atlantoaxial instability.

In dogs, the development of the axis includes fusion of seven bony elements [31]. One of these elements (centrum of proatlas) forms the apex of the dens, whereas another (centrum 1) forms the caudal part of the dens, the cranial part of the body and a smaller cranial part of the left and right cranial articular surfaces. In dog 2, an irregularly depressed area was noted in the normal position of the dens and both cranial articular surfaces were found to be smaller than normal. These observations indicate that all bone tissue originating from both centrum of proatlas and centrum 1 was missing in this dog, not only the bone tissue that should have formed the dens.

A slightly elevated body temperature was observed following cervical spinal cord injury in dogs 1 and 2. In dog 1, a distended bladder was also palpated indicating a neurogenic failure of bladder contraction. Expressed urine contained blood, but was not cultured so a urinary tract infection cannot be ruled out. In humans with traumatic spinal cord injury, episodes of fever are common and are frequently due to urinary tract infections or pneumonia [32]. Similar findings could be expected in dogs. On the other hand, humans with cervical spinal cord injuries are at risk of autonomic dysfunction including thermo-dysregulation and pyrexia (quad fever), which may be fatal [33]. Although the authors of the present paper are not aware of such descriptions in dogs, quad fever could be considered in dogs with cervical spinal cord injuries with unexplained pyrexia.

Presence or absence of deep pain sensation and findings on magnetic resonance (MR) images are significant information in prognostic evaluation of dogs with an acute-onset of tetraplegia/tetraparesis secondary to atlantoaxial subluxation [16,34,35]. Nevertheless, it may be difficult to make a certain prognosis and thereby hard to recommend euthanasia versus surgical treatment and rehabilitation of such dogs. In a study of nonsurgical treatment of atlantoaxial subluxation in 19 dogs, Havig *et al.*[36] found that a good long-term outcome was associated with an acute duration of clinical signs, but not with degree of spinal cord dysfunction at admission, radiographic appearance of the dens, age, or a history of trauma. In a prospective study including MR images of 100 human patients with a traumatic cervical spinal cord injury, Miyanji *et al.*[37] found that hemorrhage, swelling and maximum compression of the spinal cord were associated with a poor prognosis for neurologic recovery. In a one-year-old toy poodle dog presented for acute-onset tetraplegia and given a guarded prognosis for full neurological recovery, Kent *et al.*[34] chose conservative treatment with external coaptation for eight days before re-examining the dog and evaluating the prognosis. In the present study, both dogs showed irreversible lesions in the atlantoaxial region of the spinal cord consisting of necrosis, loss of neural parenchyma and infiltration of gitter cells. Dog 1 was euthanased three days and eighteen hours after the inciting incident. The spinal cord lesion of this dog was extensive, affecting a large proportion of both white and grey matter, making recovery very unlikely. Although the lesions in dog 2 affected a smaller area of the spinal cord, a considerable area of both grey and white matter was affected also in this dog. It is uncertain, but considered unlikely, that dog 2 could have recovered based on the histological findings.

In the present study, a normal dens was found in both parents and in all littermates of dog 1 while an absence or hypoplasia of the dens was found in six out of the eighteen examined relatives of dog 2. The total number of seven poodle dogs with an abnormal dens represented both sexes (3 males and 4 females) and three generations of dogs. The coefficient of relationship between dog 2 and her relatives with an abnormal dens varied from 0.5 (mother, full sibling) to 0.125 (half niece/nephew). Hence, there is good evidence to suggest that absence or hypoplasia of the dens in Standard poodle dogs is inherited in an autosomal way, but the underlying mechanisms producing the changes are unknown.

## Conclusions

Atlantoaxial subluxation with severe cervical spinal cord injury is diagnosed in young large breed dogs with an absence or hypoplasia of the dens. The subluxation may occur without a history of external trauma and may not be detectable on plain lateral or ventrodorsal radiographs. The cervical spinal cord injury will not necessarily be accompanied by signs of pain or prevent the dog to raise or move its head.

In Standard poodle dogs, an absence or hypoplasia of the dens is found in related animals and in both sexes. An autosomal manner of inheritance is presumed as causal factor. Possible clinical signs related to an absence of the dens will appear at an early age and cover momentary tetraparesis to continued tetraparesis caused by severe spinal cord injury.

## Consent

Written informed consent was obtained from the owners for publication of the case presentations and accompanying images. A copy of the written consent is available for review by the Editor-in-Chief of this journal.

## Competing interests

The authors declare that they have no competing interests.

## Authors’ contributions

ØS carried out the clinical examination of dog 2, performed the radiographic study and is the main author of the paper. HJJ did the clinical examination of dog 1, MA carried out the postmortem examination of dog 1 and RS did the postmortem examination of dog 2. All authors read and approved the final manuscript.
